# Positive-Unlabeled Learning for Pupylation Sites Prediction

**DOI:** 10.1155/2016/4525786

**Published:** 2016-08-07

**Authors:** Ming Jiang, Jun-Zhe Cao

**Affiliations:** ^1^School of Electronic Engineering, Dongguan University of Technology, Dongguan 523808, China; ^2^School of Control Science and Engineering, Dalian University of Technology, Dalian 116024, China

## Abstract

Pupylation plays a key role in regulating various protein functions as a crucial posttranslational modification of prokaryotes. In order to understand the molecular mechanism of pupylation, it is important to identify pupylation substrates and sites accurately. Several computational methods have been developed to identify pupylation sites because the traditional experimental methods are time-consuming and labor-sensitive. With the existing computational methods, the experimentally annotated pupylation sites are used as the positive training set and the remaining nonannotated lysine residues as the negative training set to build classifiers to predict new pupylation sites from the unknown proteins. However, the remaining nonannotated lysine residues may contain pupylation sites which have not been experimentally validated yet. Unlike previous methods, in this study, the experimentally annotated pupylation sites were used as the positive training set whereas the remaining nonannotated lysine residues were used as the unlabeled training set. A novel method named PUL-PUP was proposed to predict pupylation sites by using positive-unlabeled learning technique. Our experimental results indicated that PUL-PUP outperforms the other methods significantly for the prediction of pupylation sites. As an application, PUL-PUP was also used to predict the most likely pupylation sites in nonannotated lysine sites.

## 1. Introduction

Recently, a prokaryotic ubiquitin-like protein (Pup) has been identified in prokaryotes [1, 2]. Pup is an intrinsically disordered protein with 64 amino acids and marks the target proteins which are needed to be degraded [3, 4]. The process of Pup linking substrate lysine by isopeptide bonds is named pupylation which plays an important role in regulating protein degradation and signal transduction in prokaryotic cells [5]. Although pupylation and ubiquitylation are functional analogues, the enzymology involved in them is different [6]. In contrast to ubiquitylation requiring three enzymes E1 (activating enzyme), E2 (conjugating enzyme), and E3 (protein ligase), pupylation requires only two enzymes: the deamidase of Pup (DOP) and the proteasome accessory factor A (PafA) [7].

To understand the molecular mechanisms of pupylation, it is important to identify pupylation substrates and sites accurately. As the large-scale proteomics methods [8–11] are usually time-consuming and labor-intensive, several computational methods have been developed to predict the pupylation sites in recent researches. Liu et al. had developed the first predictor GPS-PUP for the prediction of the pupylation sites on the basis of group-based prediction system (GPS) 2.2 algorithm [12]; Tung developed a predictor, iPUP, by using SVM algorithm and the composition of* k*-space amino acid pairs (CKSAAPs) feature [13]; Chen et al. also proposed SVM-based predictor named PupPred, in which amino acid pairs feature was employed to encode lysine-centered peptides [14]. Recently, Hasan et al. introduced a Profile-Based Composition of* k*-Spaced Amino Acid Pairs for the prediction of protein pupylation sites and built a web server named pbPUP [15].

Note that in the aforementioned three existing computational methods, the experimentally annotated pupylation sites are used as the positive training set and the remaining nonannotated lysine residues are used as the negative training set to build classifiers for prediction of new pupylation sites from the unknown proteins. However, due to the limitations of experimental condition and technique, the remaining nonannotated lysine residues may contain some pupylation sites which are not experimentally validated yet [13, 14]. Thus, the classifiers are actually trained on a noisy negative set. As a result, the performance of the classifiers may not be as good as it was supposed to be.

In contrast to existing prediction methods, experimentally annotated pupylation sites were used as the positive training set and the remaining nonannotated lysine residues were used as the unlabeled training set in this study. We developed a novel method to predict pupylation sites by using the positive-unlabeled (PU) learning technique. This method was called PUL-PUP (PU learning for pupylation sites prediction). Experimental results show that the performance of our method significantly outperforms the other methods on both training and test sets. As an application, the most likely pupylation sites were predicted in nonannotated lysine sites by the method we proposed in this paper. PUL-PUP Matlab software package is freely accessible at https://pul-pup.github.io/.

## 2. Materials and Methods

### 2.1. Dataset

Tung's training set and independent test set [13] were used in this study. The training set consisted of 162 proteins with 183 experimentally annotated pupylation sites and 2258 nonannotated pupylation sites; the independent test set consisted of 20 proteins with 29 experimentally annotated pupylation sites and 408 nonannotated pupylation sites. Sliding window method was used to encode every lysine residue K of dataset because pupylation only occurred in lysine residues K. According to [13], window size was selected as 21 in our study.

### 2.2. Feature Extraction and Feature Selection

The CKSAAP encoding has been widely used to various posttranslational modifications' site prediction [16–18]. The CKSAAP features [13, 19] with *k* = 0, 1, 2, 3, and 4 were used to encode each residue of lysine fragment in this study. Thus, each sample was represented by 2205 features. In Tung's paper [13], chi-square test and backward feature elimination algorithm were used to remove the irrelevant and redundant features. Firstly, chi-square test was employed to rank the importance of the 2205 features. Then, the backward feature selection algorithm was used to eliminate 50 features with the lowest ranks in each iteration. Here, the top 150 CKSAAP features were selected as optimal feature set which were also same as Tung's paper [13].

### 2.3. Development of PUL-PUP

The experimentally annotated pupylation sites were used as the positive training set and the remaining nonannotated lysine residues were used as the unlabeled training set to build classifier in this study. In this way, two types of subset were received in the training set: (1) the positive dataset *P* and (2) the unlabeled dataset *U*. Thus our problem became learning from positive and unlabeled samples. We proposed a novel PU learning algorithm named PUL-PUP to predict pupylation sites. The core learning algorithm of PUL-PUP is support vector machine (SVM) which has been widely used in various biological problems [20–22]. The flowchart of PUL-PUP algorithm is shown as follows:

*Input*

(i) positive training data *P*
(ii) unlabeled data *U*

*Output*

(i) final classifier *f*

**Stage 1 (selection of initial reliable negatives).**

(i) ${RN}^{0}=\arg\max_{\begin{array}{c} N\subset U,\;|N|=|P| \end{array}}d(N,P)$

**Stage 2 (expansion of reliable negative example set).**

(i) *i* = 0;
(ii) Repeat
(iii) *U* = *U*∖RN^*i*^;
(iv) Construct two-class SVM *f* ^*i*^ based on P and RN^*i*^;
(v) Classify *U* by *f* ^*i*^;
(vi) *N*_pred_^*i*^ is the predicted negative set, where |*N*_pred_^*i*^ | ≤ 2*∗* | *P*| and *f* ^*i*^(*N*_pred_^*i*^)<−0.25;
(vii)  ${RN}^{i+1}=N_{pred}^{i}\cup N_{sv}^{i}\cup {\tilde{N}}_{sv}^{i}$ where *N*_sv_^*i*^ is the negative SVs of *f* ^*i*^, ${\tilde{N}}_{sv}^{i}$ is the surrounding points of *N*_sv_^*i*^ in *N* ^*i*^ and $\left|N_{sv}^{i}|=|{\tilde{N}}_{sv}^{i}\right|$;
(viii) *i* = *i* + 1;
(ix) until |*U* | ≤ 4*∗* | *P*|;

**Stage 3 (acquisition of final classifier).**

(i) A final SVM classifier *f* was trained on positive set *P* and representative reliable negative set RN

There are three stages in PUL-PUP algorithm as follows.

**Stage 1 (selection of initial reliable negatives).**

PUL-PUP selected the initial reliable negative set RN^0^ from unlabeled set* U* by maximum distance rule. RN^0^ should be located as far away from* P* as possible to ensure that the reliable negative set was the most dissimilar from the positive set* P*. Therefore, RN^0^ would satisfy the formula described below:

(1) RN0=argmaxN⊂UN=P⁡dN,P,

where *d*(*N*, *P*) is Euclidean distance between *N* and *P*:

(2) $$\begin{array}{c} d\left(\;N,P\;\right)=\underset{p\in P}{\min}\overset{}{\underset{n\in N}{\sum }}\left\|\;n-p\;\right\|. \end{array}$$

**Stage 2 (expansion of reliable negative example set).**

After the selection of initial reliable negative set, PUL-PUP algorithm iteratively trained a series of two-class SVM classifiers and gradually extended reliable negative set. Specifically, at the *i*th iteration, an SVM classifier *f* ^*i*^ was firstly trained in positive set *P* and current reliable negative training set RN^*i*^; then, *f* ^*i*^ would be used to classify the current unlabeled set *U* ^*i*^ and calculate its decision value. To guarantee the reliability of the negative set, samples with the decision value less than a threshold (*T*) were selected as newly predicted negatives *N*_pred_^*i*^; here *T* was set to −0.25. To overcome the problem of imbalance during the iteration, the negative support vectors *N*_sv_^*i*^ and their surrounding points in RN^*i*^, named ${\tilde{N}}_{sv}^{i}$, were used to represent the existing negative set RN^*i*^, and the size of *N*_pred_^*i*^ was controlled less than 2*∗*|*P*|. At the *i* + 1th iteration, *U* ^*i*+1^ = *U* ^*i*^∖*N*_pred_^*i*^; ${RN}^{i+1}=N_{pred}^{i}\cup N_{sv}^{i}\cup {\tilde{N}}_{sv}^{i}$. Classifier *f* ^*i*+1^ was trained in positive set *P* and current reliable negative training set RN^*i*+1^. As this process continues, RN^i^ may contain more and more false positive examples; therefore, iteration should be terminated at some point. Iteration was repeated until the size of *U* ^*i*^ goes below a threshold *r∗*|*P*|; here *r* was set to 4.

**Stage 3 (acquisition of final classifier).**

After the extraction of representative reliable negative set, a final SVM classifier *f* was trained on positive set *P* and representative reliable negative set RN.

### 2.4. SVM Parameter Selection

The core learning algorithm of PUL-PUP is support vector machine (SVM) with radial basis function (RBF) kernel. Libsvm [23] was used for training SVM models, and the grid search method was applied to tune the parameters in cross-validation. Parameter *C* was selected from {0.1, 0.2, 0.5, 1, 2, 5, 10, 20, 50, 100, 200, 500, 1000, 2000, 5000,10000}; and kernel parameter *γ* was selected from {0.00001, 0.00002, 0.00005, 0.0001, 0.0002,0.0005, 0.001, 0.002,0.005,0.01,0.02,0.05,0.1,0.2,0.5,1}. The parameters of SVM were fixed during the expansion of reliable negative example set.

### 2.5. Performance Evaluation of PUL-PUP

Five widely accepted measurements, including sensitivity (Sn), specificity (Sp), accuracy (ACC), Matthew's correlation coefficient (MCC), and area under receiver operating characteristic curve (AUC), were used to evaluate prediction performances of PUL-PUP. They are defined as

(3) Sn=TPTP+FN,Sp=TNTN+FP,ACC=TP+TNTP+FP+TN+FN,MCC=TP×TN−FN×FPTP+FN×TN+FP×TP+FP×TN+FN,

where TP, TN, FP, and FN denote the number of true positives, true negatives, false positives, and false negatives, respectively.

## 3. Results and Discussions 

### 3.1. Performance of 10-Fold Cross-Validation on Training Set

In order to evaluate the effectiveness of the selected representative reliable negative samples on pupylation sites prediction, we compared our method with two other methods including SVM_balance and PSoL [24] on training set because the core learning algorithm of our method was SVM and our method was inspired by PSoL. For PUL-PUP and PSoL algorithms, the nonannotated lysine sites were used as the unlabeled training samples. The 10-fold cross-validation of them was performed on positive set *P* and representative reliable negative set RN. For SVM_balance, a balanced negative training set which had the same size with the positive training set was randomly selected from the nonannotated lysine sites and the 10-fold cross-validation was also performed on the positive training set and the balanced negative training set to find the best parameters of SVM. The 10-fold cross-validation of the four methods was shown in Table 1. As shown in Table 1, PUL-PUP reached the highest Sn, Sp, ACC, MCC, and AUC values of 82.24%, 91.57%, 88.92%, 0.74, and 0.92, respectively, on training dataset. As the selected representative reliable negative samples, the PUL-PUP achieved an excellent performance on training set.

### 3.2. Comparison of PUL-PUP with Other Methods on Independent Test Set

To further evaluate the performance of pupylation sites prediction by PUL-PUP, we firstly compared it with PSoL and SVM_balance on independent test set. The compared results of different methods are shown in Table 2. Although SVM_balance can avoid the imbalanced problem, the performance of SVM_balance cannot be as good as the PUL-PUP because the negative training set in SVM_balance is randomly selected and cannot truly reflect the distribution of negative set well. It should be pointed out that stage 2 of PUL-PUP was similar to the negative set expansion in PSoL. But, in PUL-PUP, RN^*i*^ was represented by $N_{sv}^{i}\cup {\tilde{N}}_{sv}^{i}$ rather than *N*_sv_^*i*^ merely. Thus, more information in RN^*i*^ is included and makes our algorithm more effective than PSoL.

We also compared our method with three existing pupylation sites predictors: GPS-PUP [12], iPUP [13], and pbPUP [15] on independent test set. Three thresholds of “High,” “Medium,” and “Low” were defined for PUL-PUP according to the SVM scores which were higher than 0.9672, 0.4032, and 0.1088, respectively. The performances of PUL-PUP and three existing pupylation sites predictors were shown in Table 3. As we can see from Table 3, the performance of our algorithm outperformed the existing three predictors significantly. Taking threshold “Medium,” for example, the MCC of PUL-PUP (0.24) was higher than that of GPS-PUP (0.14), iPUP (0.16), and pbPUP (0.07). Moreover, PUL-PUP achieved the highest AUC value (0.77). As our classifier is iteratively trained on the positive and reliable negative set in this paper, the performance of our algorithm outperformed the existing three predictors significantly. This demonstrates that PUL-PUP is more suitable for predicting the pupylation sites than other methods.

### 3.3. Prediction of the Most Likely Pupylation Sites in Nonannotated Lysine Sites

For the 183 pupylated proteins in PupDB [6], there are 212 experimentally annotated pupylation sites and 2666 nonannotated lysine sites. As mentioned earlier, those nonannotated lysine sites may contain some pupylation sites which have not been experimentally validated yet. To predict the most likely pupylation sites in nonannotated lysine sites, we run PUL-PUP algorithm on all data of the PupDB. The top 20 most likely pupylation sites in nonannotated lysine sites were listed in Supplementary S1 (see Supplementary Material available online at http://dx.doi.org/10.1155/2016/4525786). Here, we just give a possible hypothesis; whether those sites will cause pupylation or not remains to be experimentally verified.

## 4. Conclusions

In this study, we have developed novel pupylation sites prediction method PUL-PUP by using the PU learning. To the best of our knowledge, this is the first time PU learning has been applied to predict the pupylation sites. Experimental results have shown that our method outperformed the existing pupylation sites predictors significantly. Moreover, the most likely pupylation sites were predicted in nonannotated lysine sites by using PUL-PUP. We believe that our method can also be applied to predict the other types of posttranslational modification sites. In future research, we will develop a web server for the PUL-PUP.

## Supplementary Material

The ‘Uniprot_AC' means the accession number of protein in Uniprot database; ‘Site' means lysine sites in the protein; the ‘Sore' means SVM sore, and the higher SVM score indicates more reliable pupylation site.

## Figures and Tables

**Table 1:** 10-fold cross-validation performance of PUL-PUP, PSoL, SVM, and SVM_balance.

Method	Sn (%)	Sp (%)	ACC (%)	MCC	AUC
PUL-PUP	82.24	91.57	88.92	0.74	0.92
PSoL	67.50	73.60	70.55	0.42	0.80
SVM_balance	76.71	63.65	69.88	0.40	0.77

**Table 2:** Independent test performance of PUL-PUP, PSoL, SVM, and SVM_balance.

Method	Sn (%)	Sp (%)	ACC (%)	MCC	AUC
PUL-PUP	68.97	70.83	70.71	0.22	0.77
PSoL	51.72	73.14	71.62	0.13	0.74
SVM_balance	62.07	67.40	67.05	0.15	0.70

**Table 3:** Independent test performance of PUL-PUP and three existing pupylation sites predictors.

Method	Threshold	Sn (%)	Sp (%)	ACC (%)	MCC	AUC
GPS-PUP	High	31.03	89.46	85.62	0.16	0.60
	Medium	34.48	85.54	82.19	0.14	
	Low	41.38	76.72	74.43	0.10	

iPUP	High	48.28	82.84	80.55	0.20	0.66
	Medium	51.72	76.47	74.83	0.16	
	Low	55.17	72.06	70.94	0.15	

pbPUP	High	17.24	88.48	83.75	0.04	0.60
	Medium	31.03	80.15	76.89	0.07	
	Low	41.38	69.85	67.96	0.07	

PUL-PUP	High	51.72	83.33	81.24	0.22	0.77
	Medium	65.52	76.72	75.97	0.24	
	Low	68.97	72.79	72.54	0.23	
