# Supplementary material for: Positive-Unlabeled Learning for Pupylation Sites Prediction
Source: Biomed Res Int. 2016 Aug 7;2016:4525786. doi: 10.1155/2016/4525786 (PMC4992543; doi:10.1155/2016/4525786)
Supplement: Supplementary file 1 — The ‘Uniprot_AC' means the accession number of protein in Uniprot database; ‘Site' means lysine sites in the protein; the ‘Sore' means SVM sore, and the higher SVM score indicates more reliable pupylation site. [file 4525786.f1.doc]

**Supplementary S1.** Top 20 most likely pupylation sites in non-annotated lysine sites

| **Uniprot_AC** | **Site** | **Sore^1^** | **Uniprot_AC** | **Site** | **Sore** |
| --- | --- | --- | --- | --- | --- |
| P04805 | 394 | 2.74 | P25437 | 26 | 2.30 |
| P0A7V8 | 151 | 2.57 | A0R5R5 | 15 | 2.29 |
| P0A7V8 | 150 | 2.57 | P00961 | 475 | 2.28 |
| A0QZ54 | 536 | 2.53 | P0A520 | 166 | 2.20 |
| P27302 | 394 | 2.47 | O05814 | 495 | 2.20 |
| P0A556 | 692 | 2.42 | A0QXX7 | 564 | 2.17 |
| P0A7V8 | 148 | 2.32 | P16659 | 568 | 2.15 |
| A0QZ54 | 531 | 2.32 | P25437 | 27 | 2.15 |
| P63345 | 527 | 2.32 | P0A6F5 | 34 | 2.15 |
| A0QQH7 | 198 | 2.31 | P0A6F5 | 42 | 2.15 |

**^1^** The higher SVM score indicates more reliable pupylation site
